# Confirmation of *Leptobrachellaventripunctata* (Fei, Ye, and Li, 1990), based on molecular and morphological evidence in Thailand

**DOI:** 10.3897/BDJ.9.e74097

**Published:** 2021-10-14

**Authors:** Yun-He Wu, Parinya Pawangkhanant, Jin-Min Chen, Wei Gao, Chatmongkon Suwannapoom, Jing Che

**Affiliations:** 1 State Key Laboratory of Genetic Resources and Evolution, Kunming Institute of Zoology, Chinese Academy of Sciences, Kunming, Yunnan, China State Key Laboratory of Genetic Resources and Evolution, Kunming Institute of Zoology, Chinese Academy of Sciences, Kunming Yunnan China; 2 Division of Fishery, School of Agriculture and Natural Resources, University of Phayao, Phayao, Thailand Division of Fishery, School of Agriculture and Natural Resources, University of Phayao Phayao Thailand; 3 Anhui Provincial Key Laboratory of the Conservation and Exploitation of Biological Resources, College of Life Sciences, Anhui Normal University, Anhui, China Anhui Provincial Key Laboratory of the Conservation and Exploitation of Biological Resources, College of Life Sciences, Anhui Normal University Anhui China

**Keywords:** Megophryidae, national new record, Chiang Rai Province, 16S rRNA

## Abstract

**Background:**

Thailand is considered a global biodiversity hotspot that is known to harbour a striking diversity of endemic species. However, several research studies have determined that the level of amphibian diversity in the country has been significantly underestimated. The megophryid genus *Leptobrachella* Smith, 1925 is currently known to include 89 species that are primarily distributed throughout southern China and Southeast Asia; however, only seven species have been found in Thailand.

**New information:**

Based on an integrative approach encompassing genetic and morphological analyses, we have concluded that the population identified from Chiang Rai Province of Thailand is conspecific with *Leptobrachellaventripunctata* (Fei, Ye, and Li, 1990). Importantly, this is the first confirmation record of this species, based on molecular and morphological evidence in Thailand. The discovery of this species reaffirms that the diversity within the genus has been underestimated with many species yet to be discovered. In addition, the findings of our study further highlight the lack of existing knowledge on amphibian taxonomy and an underestimation of the biodiversity that exists along these national border areas.

## Introduction

As a result of the increasing amounts of attention and effort devoted to herpetological research studies in Thailand over the past decade, the present knowledge of Thailand’s amphibian fauna has grown rapidly with newly-described taxa and the addition of new records from the country (e.g. Poyarkov et al. 2018, Suwannapoom et al. 2017, Suwannapoom et al. 2016, Wu et al. 2019, Yan et al. 2016). Currently, Thailand is known to be home to 194 amphibians, while at least 29 species are considered as being endemic (Poyarkov et al. 2021). However, many regions of the country, particularly along the northern borders of Thailand, have not yet been surveyed in full detail. Perhaps the most notable of these regions would be the tropical region along the Thailand-Myanmar border in Doi Tung, Chiang Rai Province.

The frog genus *Leptobrachella* Smith, 1925 is a key component of the Southeast Asian herpetofauna, which currently includes 89 species mainly being distributed throughout southern China, north-eastern India, Indochina, Malaya, Borneo and Natuna Island (Chen et al. 2018, Frost 2021). Frogs of this genus are small in size and inhabiting forest floors and rocky streams in hilly evergreen forests. Furthermore, frogs of this genus are often difficult to find and identify down to the species level due to their inconspicuous and morphological conservatism. However, recent increases in survey efforts, along with the use of updated molecular and acoustic data, have revealed an underestimation of the taxonomic diversity of the genus. These new efforts have established descriptions of many new species, while also yielding some newly-recorded species (e.g. Chen et al. 2018, Stuart and Rowley 2020, Yuan et al. 2017). According to Poyarkov et al. (2021), nine species of the genus *Leptobrachella* are distributed in Thailand, which include *L.fuliginosa*, *L.heteropus*, *L.melanoleuca*, *L.minima*, *L.pelodytoides*, *L.sola*, *L.bourreti*, *L.ventripunctata* and *L.zhangyapingi*. However, records of *L.bourreti* for Thailand seem to be based on misidentifications and need to be verified by further studies (Poyarkov et al. 2021, Frost 2021). There is no evidence of records for *L.ventripunctata* in Thailand (Poyarkov et al. 2021). Thus, only seven species of this genus are currently undisputed in Thailand.

The species *Leptobrachellaventripunctata* was originally described from Mengla County, Yunnan, China (Fei et al. 1990). Recently, relevant research studies have observed new distributions of this species. For example, Luong et al. (2019) reported distribution of this species in Dien Bien Province, Vietnam. In addition, Chen et al. (2018) recorded this species in Caiyanghe, Yunnan, China, as well as localities in Tuyen Quang, Thanh Hoa, Cao Bang, Lao Cai and Phu Tho Provinces in Vietnam. To date, this species is known to be distributed widely throughout southern Yunnan in China, Phongsaly in Laos and in the Son La, Vinh Phuc, Thanh Hoa, Tuyen Quang, Cao Bang and Dien Bien Provinces of northern Vietnam (Frost 2021).

During recent field surveys, conducted in Chiang Rai Province of northern Thailand in 2017, we collected one specimen that we have assigned to the genus *Leptobrachella*, based on morphological characteristics. Subsequent detailed morphological comparisons and phylogenetic analyses indicate that the newly-identified species in Chiang Rai Province should be assigned to *L.ventripunctata*. Therefore, we have further confirmed the presence of *L.ventripunctata* in Thailand.

## Materials and methods

### Sampling

**Figure 1.**
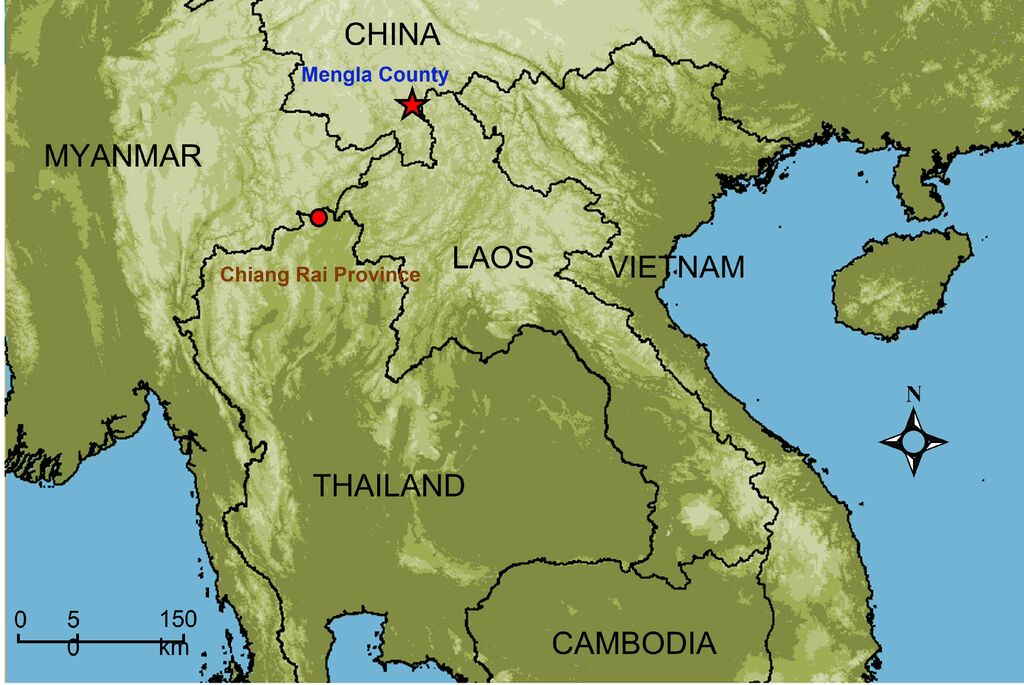
Map showing the new record in Thailand (red circle) and the type locality of *L.ventripunctata* (red star) in China.

Field work was conducted in the environs of Doi Tung, Chiang Rai Province, Thailand (20°19'36.1"N, 99°49'35.0"E, 650 m a.s.l.) and one specimen was collected by Chatmongkon Suwannapoom on 16 July 2017 (Fig. 1). The specimen was photographed in situ. The specimen was euthanised using benzocaine, then liver tissue was extracted, which was stored in 95% ethanol. The voucher specimen was fixed with 10% formalin and later stored in 70% ethanol. The voucher specimen and tissue sample were then deposited in the herpetological collections of the School of Agriculture and Natural Resources, University of Phayao (AUP), Phayao, Thailand.

### Molecular analysis

Genomic DNA was extracted from the liver tissue sample using standard phenol-chloroform protocols (Sambrook et al. 1989). A partial fragment of the mitochondrial gene 16S rRNA (16S) was amplified and sequenced using the following primers: 16SAR (5'-CGCCTGTTTAYCAAAAACAT-3'; Kocher et al. 1989) and 16SBR (5'-CCGGTYTGAACTCAGATCAYGT-3'; Kocher et al. 1989). Amplification was performed in a 25 µl volume reaction according to the following procedure: initial denaturation at 95°C for 5 min, 35 cycles of denaturation at 95°C for 1 min, annealing at 55°C for 1 min, extension at 72°C for 1 min and a final extension at 72°C for 10 min. PCR products were purified using a Gel Extraction Mini Kit (A T G C, Bangkok, Thailand). All sequencing was conducted on an ABI PRISM 3730 automated sequencer (Applied Biosystems, Foster City, CA, USA). The new sequence was first assembled and edited using AutoSeqMan (Sun 2018).

To study the existing phylogenetic relationships amongst *Leptobrachella*, phylogenetic trees were reconstructed, based on the partial mitochondrial 16S rRNA gene. Homologous sequences of the related species in the genus *Leptobrachella* and those of the outgroups *Megophrysglandulosa* (KIZ048439) and *Leptobrachiumhuashen* (KIZ049025) (Chen et al. 2018) were downloaded from GenBank (Table 1). All sequences were aligned using MUSCLE 3.6 (Edgar 2004), visually checked for accuracy and then trimmed to minimise missing characters in MEGA v.6.0.6 (Tamura et al. 2013).

Phylogenetic reconstructions using Bayesian Inference (BI) and Maximum Likelihood (ML) were executed in the CIPRES web server (Miller et al. 2010). Data were tested in JMODELTEST 2.1.7 (Darriba et al. 2012) using Bayesian Information Criteria to provide the best-fitting nucleotide substitution models (BIC; Posada 2008). For BI analyses, two separate runs were performed with four Markov chains using the GTR+I+G model. Each run was conducted for 10 million generations, while every 100 generations were sampled with a burn-in value of 25%. Convergence was assessed by the average standard deviation of split frequencies (below 0.01) and ESS values (over 200) in TRACER 1.5 (Rambaut and Drummond 2009). ML analysis was performed using RAxML with 1,000 bootstrap replications using the rapid bootstrap feature under the GTR+G model (random seed value of 12,345) (Stamatakis 2014). Apart from the phylogenetic tree-based methods, we also calculated the degree of row pairwise sequence divergence using uncorrected p-distances and complete deletion implemented in MEGA v.6.0.6 (Tamura et al. 2013).

### Morphology

Measurements were taken using a digital caliper to the nearest 0.1 mm. Abbreviations are presented following the method employed by Matsui (1984) for 25 morphological characteristics: (1) Snout-vent length (SVL); (2) Head length (HL); (3) Head width (HW); (4) Snout length (SL); (5) Distance from the centre of the nostril to the tip of the snout (SN); (6) Nostril-eye distance (N-EL); (7) Eye diameter (ED); (8) Tympanum diameter (TD); (9) Internarial distance (IND); (10) Interorbital distance (IOD); (11) Upper eyelid width (UEW); (12) Forelimb length (FLL); (13) Lower arm length (LAL); (14) Hand length (HAL); (15) First finger length (1FL); (16) Third finger disc diameter (3FDD); (17) Outer palmar tubercle length (OPTL); (18) Inner palmar tubercle length (IPTL); (19) Tibia length (TL); (20) Foot length (FL); (21) Hind-limb length (HLL); (22) Fourth toe disc diameter (4TDD); (23) Inner metatarsal tubercle length (IMTL); (24) Outer metatarsal tubercle length (OMTL) and (25) First toe length (1TOEL).

## Data resources

### Molecular Phylogeny

**Figure 2.**
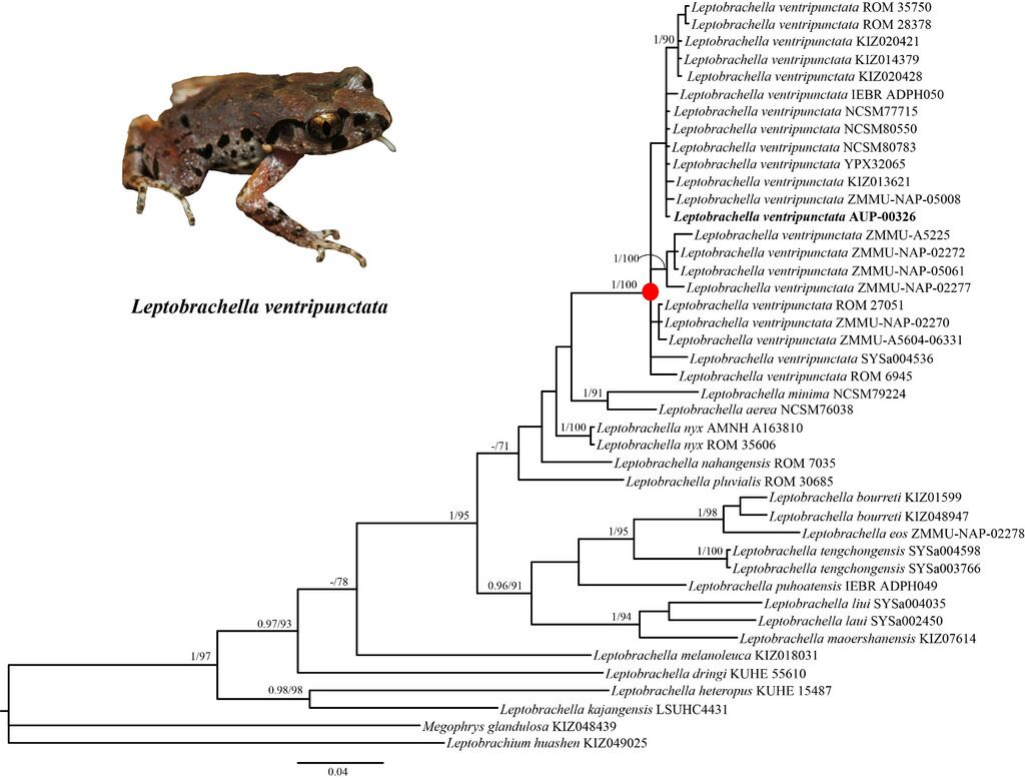
Phylogram of *Leptobrachella* resulting from the analyses of one fragment of the mitochondrial 16S gene. Nodal support values with Bayesian posterior probabilities (BPP) >= 95%/bootstrap support (BS) >= 70 are shown near the node. A “–” denotes Bayesian posterior probabilities (BPP) < 95% and bootstrap support (BS) < 70. Node values with Bayesian posterior probabilities (BPP) < 95%/bootstrap support (BS) < 70 are not shown.

The final aligned dataset of 16S nucleotide sequences contained 41 individuals with 510 bp. Amongst the 510 sites, 324 were established as conserved sites and 184 were considered variable sites, of which 127 were found to be potentially parsimony-informative sites (excluding outgroups). The Bayesian Inference (BI) and Maximum Likelihood (ML) phylogenetic trees yielded essentially identical topologies. This was true, except for the poorly-supported nodes, which have been integrated in Fig. 2. The phylogenetic analysis suggested that the newly-collected singular specimen from Chiang Rai was nested in the genus *Leptobrachella* and formed a monophyletic clade with *L.ventripunctata* obtained from China, Laos and Vietnam with strong support (Bayesian posterior probabilities (PP) = 1.00; ML bootstrap support (BS) = 100%).

**Figure 3.**
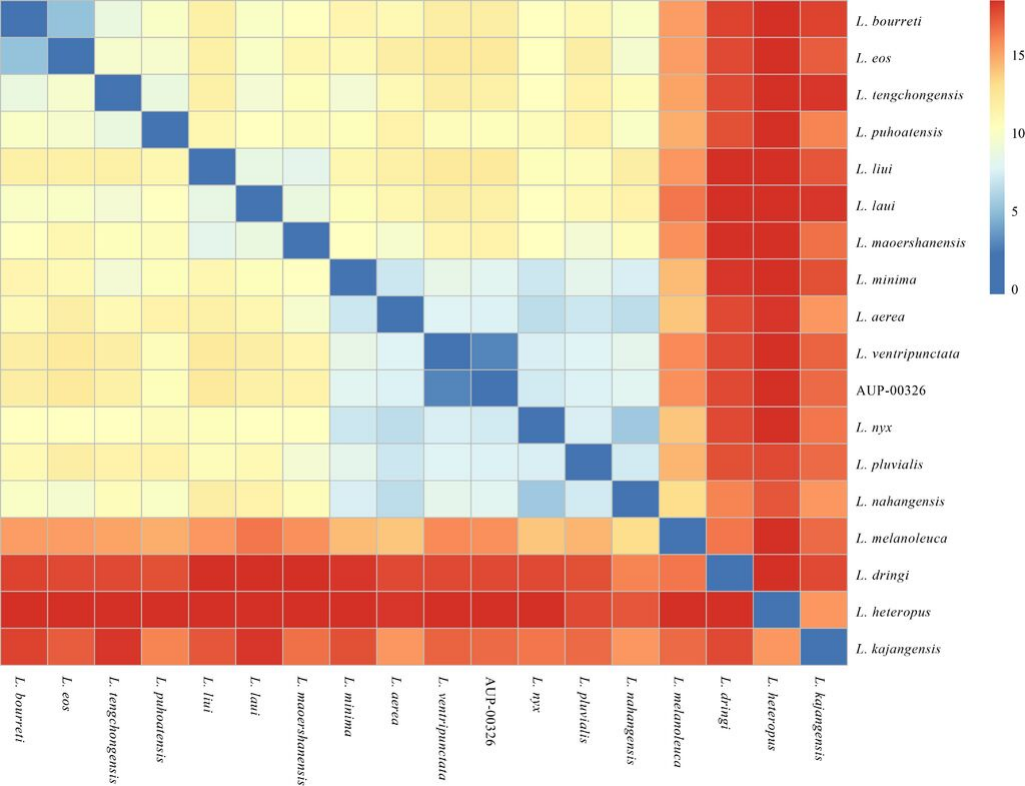
Heatmap of pairwise uncorrected p-distances of the 16S rRNA gene of *Leptobrachella* species included in phylogenetic analyses. The colours red to blue indicate high to low divergences.

Interspecific genetic divergene (uncorrected p-distance) between the new sample obtained from Chiang Rai, Thailand and the other species of *Leptobrachella* varied from 4.9% (versus *L.bourreti*) to 17.9% (versus *L.heteropus*) (Fig. 3, Suppl. material 1, Suppl. material 2). The genetic divergence between the individual specimen of *Leptobrachella*, collected from Chiang Rai and the *L.ventripunctata* specimens collected from Vietnam, Laos and China, was found to be very small (0–0.4%, Suppl. material 2).

Morphologically, the specimen from Chiang Rai shows a similar appearance with original description of *L.ventripunctata*: pupil vertical; iris distinctly bicoloured; fingers with lateral dermal fringes, absent for toes; belly creamy white with many scattered brown spots. Therefore, we determined that AUP-00326 belonged to *L.ventripunctata*.

## Taxon treatments

### Leptobrachella ventripunctata

(Fei, Ye & Li, 1990)

94F09AAF-60F0-5811-BD45-4F4132716CF1

#### Materials

- **Type status:** Other material. **Occurrence:** catalogNumber: AUP-00326; individualCount: 1; sex: male; lifeStage: adult; **Taxon:** scientificName: *Leptobrachellaventripunctata*; class: Amphibia; order: Anura; family: Megophryidae; genus: Leptobrachella; specificEpithet: *ventripunctata*; **Location:** country: Thailand; countryCode: TL; stateProvince: Chiang Rai; locality: Doi Tung; verbatimElevation: 650 m; verbatimLatitude: 20°19'36.1"N; verbatimLongitude: 99°49'35.0"E; **Record Level:** basisOfRecord: preserved specimen

#### Description

**Figure 4.**
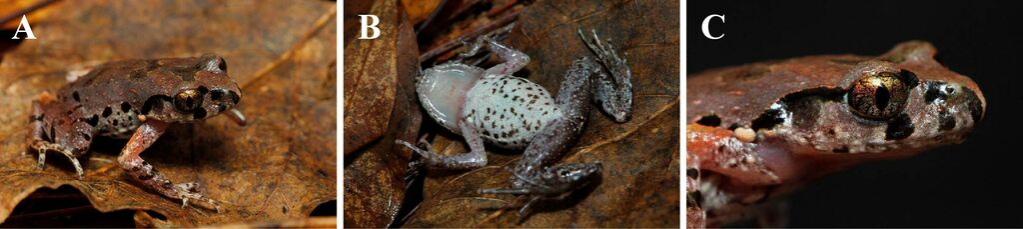
Male of *L.ventripunctata* (AUP-00326) in life. **A.** Dorsolateral view **B.** ventral view **C.** lateral view of the head. Photo by P. Pawangkhanant.

Morphological descriptions of the specimen obtained from Thailand (measurements shown in Suppl. material 3) are as follows: adult male with SVL 28.9 mm; head length (HL 13.1 mm, 45.3% of SVL) longer than width (HW 11.6 mm, 40.1% of SVL); snout slightly pointed, projecting beyond lower jaw; nostrils slightly closer to eyes than tip of snout (N-EL 2.5 mm, 8.7% of SVL; SN 2.7 mm, 9.3% of SVL); canthus rostralis distinct; loreal region concave; snout (SL 5.3 mm, 18.3% of SVL) longer than eye diameter (EL 4.6 mm, 15.9% of SVL); width of upper eyelid (UEW 4.5 mm) larger than interorbital distance (IOD 3.5 mm) and internasal distance (IND 3.1 mm); tympanum distinct, rounded, length (TD 2.4 mm, 8.3% of SVL) nearly half the diameter of the eye; vomerine teeth absent; pupil vertical; tongue pyriform, deeply notched posteriorly; supratympanic fold distinct, running from posterior corner of eye towards axilla (Fig. 4).

Forelimbs slender; relative finger lengths: I<II<IV<III; tips of all fingers rounded and slightly swollen; fingers with lateral dermal fringes; no webbing between fingers; subarticular tubercles distinct, large, near the palm smaller; two palmar tubercles, inner palmar tubercle large, rounded (IPTL 1.6 mm, 5.5% of SVL), outer palmar tubercle relatively small (OPTL 1.0 mm, 3.5% of SVL) (Fig. 4).

Hind-limbs long, foot slightly shorter than tibia, tibia length (TL) 56.1% of SVL, foot length (FL) 51.6% of SVL; tibial-tarsal articulation beyond the tip of the eye when the hind-limb is adpressed along the side of the body; heels overlapping when the ﬂexed legs are held at right angles to the body axis; relative toe length: I<II<III<IV<V; tips of toes rounded and slightly swollen; rudimentary webbing between toes; subarticular tubercles distinct, rounded, inner metatarsal tubercle distinct and oval (IMTL 0.9 mm, 3.1% of SVL), outer metatarsal tubercle distinct (1.0 mm, 3.5% of SVL) (Fig. 4).

Dorsal surfaces of head, body, thigh, tibia and flank of body appear relatively rough with numerous granular spots; ventral surfaces smooth; pectoral gland and femoral gland distinct, oval; pectoral glands larger than femoral glands; supra-axillary gland raised. Ventrolateral gland distinctly visible, forming discontinuous cream-white lines on flanks (Fig. 4).

##### Color in life

Dorsal surface appears reddish brown with small reddish tubercles; a dark inverted triangular marking in the interorbital region, a "W"-shaped marking appears between axillae; flanks scattered with some distinct moderate black blotches; upper lips with three black vertical bars; loreal and tympanic region with distinct black markings; supratympanic ridge appears reddish and lower margin of supratympanic fold appears black; elbow to upper arm and tibio-tarsal articulation distinct and reddish-brown in colour on the dorsum; dorsal surface of lower arms, legs, fingers and toes with dark brown crossbars; throat pale with some dusting; belly creamy white with many scattered brown spots; ventrolateral glands, pectoral glands and femoral glands appear white or yellowish-white; ventral surface of limbs appear grey brown with dark brown and white speckling or dots; iris distinctly bicoloured, typically bright orange-red on upper half and silvery-white on lower half (Fig. 4).

#### Distribution

This species was previously only known to be indigenous to extreme southern Yunnan, China; Phongsaly, Xiangkhouang and Houaphanh Provinces in Laos; as well as Son La, Vinh Phuc, Thanh Hoa, Tuyen Quang, Cao Bang, Nghe An, Bac Giang, Phu Tho, Hoa Binh, Lao Cai and Dien Bien Provinces in Vietnam (Chen et al. 2018, Frost 2021). Importantly, this first specific record of *L.ventripunctata* in Thailand substantially expands the known area of distribution of this species.

#### Ecology

**Figure 5.**
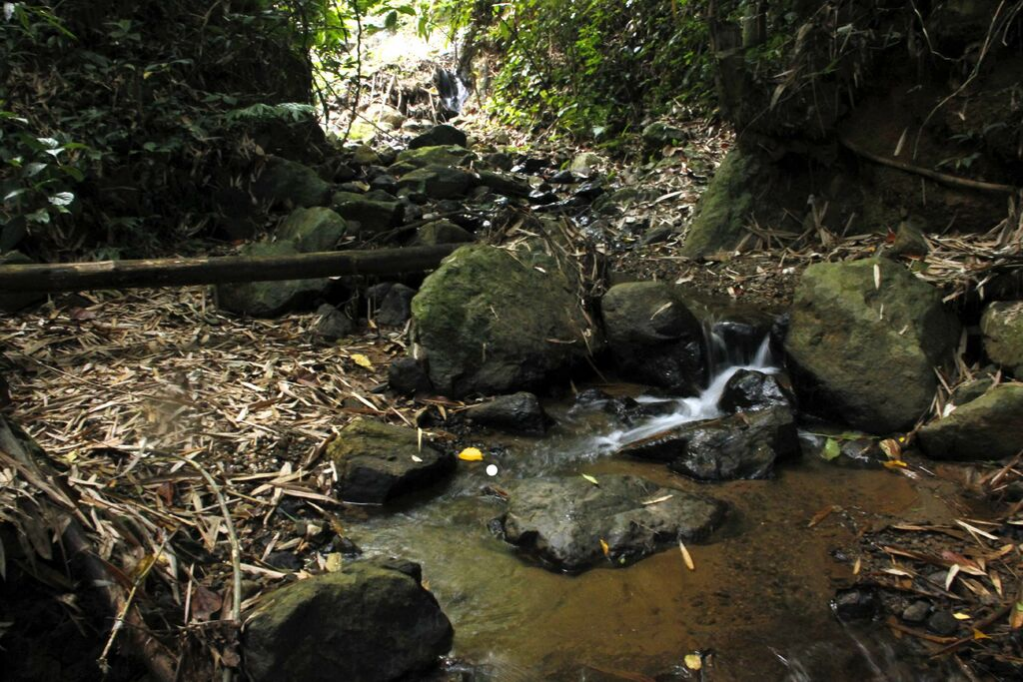
Habitat at collection site of *L.ventripunctata* in Chiang Rai Province, Thailand.

This species lives mainly in the litter on both sides of streams. An individual specimen was observed at night sitting on the branch of a shrub that was about 1-2 m off the ground in an evergreen forest surrounded by trees near a stream with nearby herbaceous plants (Fig. 5). This species has been associated with the sympatric distribution of *Micrylettainornata* and *Limnonectestaylori*.

## Discussion

Poyarkov et al. (2021) mentioned the distribution of *L.ventripunctata* in the extreme north of Thailand, but did not provide any evidence, such as the specific locality, voucher specimens or DNA sequences. This is the first record of the species with morphological data and molecular evidence.

Thailand represents an important component of the Indo-Burma biodiversity hot-spot. Its northern region lies at a biogeographic crossroads where the fauna of China, Indochina, India and Himalaya converge (Myers et al. 2000). Recently, along with an increasing intensity of field investigations and the application of new techniques, biodiversity studies in this region have expanded. Consequently, many new species and records have been described and reported (e.g. Lorphengsy et al. 2021, Wu et al. 2019). The confirmation of *L.ventripunctata* in this study increases the total number of known amphibian species in Thailand from 125 (Khonsue and Thirakhupt 2001) to 194, along with the known number of *Leptobrachella* species from seven to eight. The following species of *Leptobrachella* are known to be from Thailand: *L.ventripunctata*, *L.pelodytoides*, *L.fuliginosa*, *L.heteropus*, *L.melanoleuca*, *L.minima*, *L.sola* and *L.zhangyaping*. The discovery of this species reaffirms that, to date, the diversity within the genus has been underestimated with many species yet to be discovered in this country.

Furthermore, our study further highlights a lack of existing knowledge on amphibian taxonomy and identifies an underestimation of the potential biodiversity along these national border areas. In recent years, many new recorded species have been found in border areas, such as *Nidiranachapaensis* (Yuan et al. 2019), *Gracixalusquangi* (Lorphengsy et al. 2021) and *Thelodermapyaukkya* (Du et al. 2020), while a number of studies have called for enhanced surveys of species diversity in these border areas (Chen et al. 2020, Chen et al. 2018, Wu et al. 2020, Yuan et al. 2019). The findings of our research further support this view. The newly-recorded species we reported was found along the border of Thailand and Myanmar, which is very close to Laos. Currently, distributions of this species have been recorded in Laos and Thailand, whereas they have not been definitively reported in Myanmar (Chen et al. 2018, Frost 2021). Considering that it is located in the same zoogeographic region comprised of continuous forests habitats, we suspect that this species is also present in Myanmar. In the future, it will be important to strengthen levels of international cooperation in order to further clarify the full range of this species.

## Supplementary Material

01130BC3-9F37-535D-B23C-CFFF05125DB4 10.3897/BDJ.9.e74097.suppl1 Supplementary material 1 Mean pairwise uncorrected p-distance Data type Table Brief description The mean pairwise uncorrected p-distance (%) of 16S rRNA gene amongst the species of *Leptobrachella*. Sample ID corresponds to those in Table 1 File: oo_583031.docx https://binary.pensoft.net/file/583031 Wu et al.

43D51313-5CD8-5E22-BECE-2B47E8F95EB5 10.3897/BDJ.9.e74097.suppl2 Supplementary material 2 Average uncorrected p-distances Data type Table Brief description Average uncorrected p-distances amongst those calculated from 16S rRNA gene sequences File: oo_583032.docx https://binary.pensoft.net/file/583032 Wu et al.

FA8ECA54-0E66-50D6-B08B-4C813E06BFAD 10.3897/BDJ.9.e74097.suppl3 Supplementary material 3 Measurement and proportions Data type Table Brief description Measurement (in mm) of and proportions of *Leptobrachellaventripunctata*. File: oo_583033.docx https://binary.pensoft.net/file/583033 Wu et al.

XML Treatment for Leptobrachella ventripunctata

## Figures and Tables

**Table 1.** Localities, voucher ID and GenBank numbers for all samples used in this study.

**ID**	**Species**	**Voucher ID**	**Locality**	**16S rRNA**	**Reference**
	**Ingroup**				
1	*Leptobrachellabourreti*	KIZ01599	Jinxiu, Guangxi, China	MH055870	Chen et al. 2018
2	*Leptobrachellabourreti*	KIZ048947	Dawei Shan, Yunnan, China	MH055871	Chen et al. 2018
3	*Leptobrachellaeos*	ZMMU-NAP-02278	Vientiane, Laos	MH055880	Chen et al. 2018
4	*Leptobrachellatengchongensis*	SYS a004598	Gaoligong Shan, Yunnan, China	KU589209	Yang et al. 2016
5	*Leptobrachellatengchongensis*	SYS a003766	Gaoligong Shan, Yunnan, China	MH055897	Chen et al. 2018
6	*Leptobrachellapuhoatensis*	IEBR ADPH049	Pu Hu Nature Reserve, Thanh Hoa, Vietnam	MH055898	Chen et al. 2018
7	*Leptobrachellaliui*	SYS a004035	Wugong Shan, Jiangxi, China	MH055916	Chen et al. 2018
8	*Leptobrachellalaui*	SYS a002450	Shenzhen, Guangdong, China	MH055904	Chen et al. 2018
9	*Leptobrachellamaoershanensis*	KIZ07614	Mao’er Shan, Guangxi, China	MH055927	Chen et al. 2018
10	*Leptobrachellaminima*	NCSM 79224	Louangphrabang, Luang Prabang, Laos	MH055845	Chen et al. 2018
11	*Leptobrachellaaereus*	NCSM 76038	Vilabuly, Savannakhet, Laos	MH055809	Chen et al. 2018
12	*Leptobrachellaventripunctata*	ROM 35750	Pu'er, Yunnan, China	MH055828	Chen et al. 2018
13	*Leptobrachellaventripunctata*	ROM 28378	Sapa, Lao Cai, Vietnam	MH055829	Chen et al. 2018
14	*Leptobrachellaventripunctata*	KIZ020421	Jinuo Shan, Yunnan, China	MH055825	Chen et al. 2018
15	*Leptobrachellaventripunctata*	KIZ014379	Caiyanghe, Yunnan, China	MH055826	Chen et al. 2018
16	*Leptobrachellaventripunctata*	KIZ020428	Maandi, Yunnan, China	MH055827	Chen et al. 2018
17	*Leptobrachellaventripunctata*	IEBR ADPH050	Pu Hu Nature Reserve, Thanh Hoa, Vietnam	MH055819	Chen et al. 2018
18	*Leptobrachellaventripunctata*	NCSM 77715	Viengthong, Houaphanh, Laos	MH055820	Chen et al. 2018
19	*Leptobrachellaventripunctata*	NCSM 80550	Boun Tay, Phongsaly, Laos	MH055821	Chen et al. 2018
20	*Leptobrachellaventripunctata*	NCSM 80783	Kham, Xiangkhouang, Laos	MH055822	Chen et al. 2018
21	*Leptobrachellaventripunctata*	Tissue ID: YPX32065	Huanglianshan National Nature Reserve, Yunnan, China	MH055823	Chen et al. 2018
22	*Leptobrachellaventripunctata*	KIZ013621	Wenlong, Yunnan, China	MH055824	Chen et al. 2018
23	*Leptobrachellaventripunctata*	ZMMU-NAP-05008	Muong Nhe Nature Reserve, Dien Bien, Vietnam	MH055830	Chen et al. 2018
24	*Leptobrachellaventripunctata*	ZMMU-A-5225	Xuan Son National Park, Phu Tho, Vietnam	MH055835	Chen et al. 2018
25	*Leptobrachellaventripunctata*	ZMMU-NAP-02272	Hoa Binh, Vietnam	MH055836	Chen et al. 2018
26	*Leptobrachellaventripunctata*	ZMMU-NAP-05061	Kim Son, Nghe An, Vietnam	MH055837	Chen et al. 2018
27	*Leptobrachellaventripunctata*	ZMMU-NAP-02277	Thuong Xuan, Thanh Hoa, Vietnam	MH055838	Chen et al. 2018
28	*Leptobrachellaventripunctata*	ROM 27051	Quang Thanh Village, Cao Bang, Vietnam	MH055832	Chen et al. 2018
29	*Leptobrachellaventripunctata*	ZMMU-NAP-02270	Bac Giang, Vietnam	MH055833	Chen et al. 2018
30	*Leptobrachellaventripunctata*	ZMMU-A-5604-06331	Vinh Phuc, Vietnam	MH055834	Chen et al. 2018
31	*Leptobrachellaventripunctata*	SYS a004536	Zhushihe, Yunnan, China	MH055831	Chen et al. 2018
32	*Leptobrachellaventripunctata*	ROM 6945	Na Hang Nature Reserve, Tuyen Quang, Vietnam	MH055839	Chen et al. 2018
33	*Leptobrachellaventripunctata*	AUP-00326	Doi Tung, Chiang Rai, Thailand	**OK430887**	**This study**
34	*Leptobrachellanyx*	AMNH A163810	Mount Tay Conn Linh, Ha Giang, Vietnam	DQ283381	Frost 2021
35	*Leptobrachellanyx*	ROM 35606	Malipo, Yunnan, China	MH055814	Chen et al. 2018
36	*Leptobrachellapluvialis*	ROM 30685	Fansipan, Lao Cai, Vietnam	MH055843	Chen et al. 2018
37	*Leptobrachellanahangensis*	ROM 7035	Na Hang Nature Reserve, Tuyen Quang, Vietnam	MH055853	Chen et al. 2018
38	*Leptobrachellamelanoleuca*	KIZ018031	Kapoe, Ranong, Thailand	MH055967	Chen et al. 2018
39	*Leptobrachelladringi*	KUHE:55610	Gunung Mulu, Malaysia	AB847553	Matsui et al. 2014
40	*Leptobrachellaheteropus*	KUHE 15487	Larut, Perak, Malaysia	AB530453	Matsui et al. 2014
41	*Leptobrachellakajangensis*	LSUHC:4431	Tioman, Malaysia	LC202001	Matsui et al. 2017
	**Outgroups**				
42	*Megophrysglandulosa*	KIZ048439	Yunnan, China	KX811762	Chen et al. 2016
43	*Leptobrachiumhuashen*	KIZ049025	Yunnan, China	KX811931	Chen et al. 2016
