# Supplementary material for: Confirmation of Leptobrachellaventripunctata (Fei, Ye, and Li, 1990), based on molecular and morphological evidence in Thailand
Source: Biodivers Data J. 2021 Oct 14;9:e74097. doi: 10.3897/BDJ.9.e74097 (PMC8530995; doi:10.3897/BDJ.9.e74097)
Supplement: Supplementary material 1 — Mean pairwise uncorrected p-distance [file bdj-09-e74097-s001.docx]

**Supplementary materials 1.** The mean pairwise uncorrected p-distance (%) of 16S rRNA gene among the species of *Leptobrachella*. Sample ID corresponds to those in Table 1.

| **ID** | **Species** | **1** | **2** | **3** | **4** | **5** | **6** | **7** | **8** | **9** | **10** | **11** | **12** | **13** | **14** | **15** | **16** | **17** | **18** |
| --- | --- | --- | --- | --- | --- | --- | --- | --- | --- | --- | --- | --- | --- | --- | --- | --- | --- | --- | --- |
| **1** | *Leptobrachella bourreti* |  | 0.7 | 1.2 | 1.2 | 1.3 | 1.2 | 1.2 | 1.2 | 1.2 | 1.3 | 1.3 | 1.2 | 1.3 | 1.1 | 1.6 | 1.7 | 1.8 | 1.7 |
| **2** | *Leptobrachella eos* | 3.0 |  | 1.2 | 1.2 | 1.3 | 1.2 | 1.3 | 1.3 | 1.4 | 1.3 | 1.3 | 1.3 | 1.4 | 1.2 | 1.6 | 1.7 | 1.8 | 1.7 |
| **3** | *Leptobrachella tengchongensis* | 6.5 | 7.4 |  | 1.1 | 1.4 | 1.2 | 1.3 | 1.1 | 1.3 | 1.3 | 1.3 | 1.2 | 1.3 | 1.3 | 1.6 | 1.7 | 1.8 | 1.8 |
| **4** | *Leptobrachella puhoatensis* | 7.7 | 7.4 | 6.5 |  | 1.3 | 1.2 | 1.2 | 1.2 | 1.3 | 1.2 | 1.2 | 1.2 | 1.3 | 1.2 | 1.6 | 1.7 | 1.7 | 1.6 |
| **5** | *Leptobrachella liui* | 9.3 | 9.3 | 9.3 | 8.8 |  | 1.2 | 1.1 | 1.4 | 1.3 | 1.3 | 1.3 | 1.3 | 1.3 | 1.4 | 1.6 | 1.7 | 1.8 | 1.7 |
| **6** | *Leptobrachella laui* | 7.7 | 7.7 | 7.0 | 7.9 | 6.3 |  | 1.1 | 1.2 | 1.3 | 1.3 | 1.3 | 1.2 | 1.3 | 1.3 | 1.6 | 1.7 | 1.8 | 1.7 |
| **7** | *Leptobrachella maoershanensis* | 8.0 | 8.8 | 8.1 | 8.4 | 6.0 | 6.5 |  | 1.2 | 1.2 | 1.2 | 1.3 | 1.2 | 1.2 | 1.2 | 1.6 | 1.7 | 1.8 | 1.7 |
| **8** | *Leptobrachella minima* | 9.0 | 8.6 | 7.0 | 8.1 | 8.8 | 8.1 | 7.9 |  | 1.0 | 1.0 | 1.1 | 1.0 | 1.1 | 1.0 | 1.6 | 1.7 | 1.7 | 1.7 |
| **9** | *Leptobrachella aerea* | 8.6 | 9.5 | 8.6 | 9.1 | 9.3 | 8.8 | 7.4 | 4.7 |  | 1.0 | 1.0 | 1.0 | 1.0 | 0.9 | 1.5 | 1.7 | 1.7 | 1.6 |
| **10** | *Leptobrachella ventripunctata* | 9.4 | 9.9 | 9.5 | 8.4 | 9.9 | 9.4 | 9.0 | 6.1 | 5.6 |  | 0.2 | 1.0 | 1.0 | 1.1 | 1.6 | 1.7 | 1.7 | 1.6 |
| **11** | AUP-00326 | 9.4 | 9.8 | 9.3 | 8.1 | 9.8 | 9.3 | 9.1 | 5.8 | 5.3 | 0.6 |  | 1.0 | 1.1 | 1.1 | 1.6 | 1.7 | 1.7 | 1.6 |
| **12** | *Leptobrachella nyx* | 7.9 | 7.9 | 7.9 | 8.4 | 8.1 | 7.9 | 7.9 | 4.7 | 4.2 | 5.2 | 4.9 |  | 1.0 | 0.8 | 1.6 | 1.6 | 1.7 | 1.7 |
| **13** | *Leptobrachella pluvialis* | 8.5 | 9.5 | 9.1 | 9.1 | 8.4 | 8.6 | 7.0 | 6.0 | 4.7 | 5.6 | 5.3 | 5.1 |  | 1.0 | 1.6 | 1.7 | 1.7 | 1.7 |
| **14** | *Leptobrachella nahangensis* | 7.6 | 7.2 | 8.4 | 7.7 | 9.5 | 9.1 | 8.4 | 5.1 | 4.2 | 6.0 | 5.8 | 3.3 | 4.9 |  | 1.5 | 1.5 | 1.6 | 1.7 |
| **15** | *Leptobrachella melanoleuca* | 12.8 | 12.8 | 12.6 | 12.3 | 13.0 | 14.0 | 13.3 | 11.9 | 11.6 | 13.4 | 13.3 | 11.6 | 12.1 | 10.9 |  | 1.7 | 1.9 | 1.7 |
| **16** | *Leptobrachella dringi* | 15.6 | 15.3 | 15.3 | 15.1 | 17.0 | 16.0 | 16.5 | 15.8 | 15.3 | 15.4 | 15.3 | 15.3 | 15.1 | 13.7 | 14.0 |  | 1.8 | 1.6 |
| **17** | *Leptobrachella heteropus* | 17.1 | 17.2 | 17.2 | 16.3 | 18.6 | 18.8 | 17.4 | 16.7 | 15.8 | 16.5 | 16.5 | 16.3 | 15.3 | 14.9 | 17.0 | 16.7 |  | 1.6 |
| **18** | *Leptobrachella kajangensis* | 15.5 | 14.7 | 15.8 | 13.7 | 14.9 | 15.8 | 14.2 | 15.1 | 13.0 | 14.5 | 14.4 | 14.0 | 14.4 | 13.0 | 14.4 | 15.3 | 13.0 |  |
