# Supplementary material for: Confirmation of Leptobrachellaventripunctata (Fei, Ye, and Li, 1990), based on molecular and morphological evidence in Thailand
Source: Biodivers Data J. 2021 Oct 14;9:e74097. doi: 10.3897/BDJ.9.e74097 (PMC8530995; doi:10.3897/BDJ.9.e74097)
Supplement: Supplementary material 2 — Average uncorrected p-distances [file bdj-09-e74097-s002.docx]

**Supplementary materials 2.** Average uncorrected p-distances among the *Leptobrachella* species calculated from 16S rRNA gene sequences. Sample ID corresponds to those in Table 1.

| **ID** | **1** | **2** | **3** | **4-5** | **6** | **7** | **8** | **9** | **10** | **11** | **12-13** | **14-16** | **17-18** | **19-21** | **22** | **23** | **24** | **25-26** | **27** | **28-29** | **30** | **31** | **32** | **33** | **34-35** | **36** | **37** | **38** | **39** | **40** | **41** |
| --- | --- | --- | --- | --- | --- | --- | --- | --- | --- | --- | --- | --- | --- | --- | --- | --- | --- | --- | --- | --- | --- | --- | --- | --- | --- | --- | --- | --- | --- | --- | --- |
| **1** |  | 0.5 | 0.8 | 1.1 | 1.2 | 1.3 | 1.2 | 1.3 | 1.3 | 1.3 | 1.3 | 1.4 | 1.3 | 1.3 | 1.3 | 1.3 | 1.4 | 1.4 | 1.3 | 1.3 | 1.3 | 1.3 | 1.3 | 1.3 | 1.3 | 1.3 | 1.2 | 1.5 | 1.6 | 1.8 | 1.7 |
| **2** | 1.2 |  | 0.8 | 1.2 | 1.3 | 1.4 | 1.2 | 1.2 | 1.3 | 1.3 | 1.3 | 1.3 | 1.3 | 1.3 | 1.3 | 1.3 | 1.4 | 1.4 | 1.3 | 1.3 | 1.3 | 1.3 | 1.3 | 1.3 | 1.2 | 1.3 | 1.2 | 1.5 | 1.7 | 1.7 | 1.7 |
| **3** | 2.8 | 3.3 |  | 1.3 | 1.2 | 1.4 | 1.2 | 1.3 | 1.3 | 1.4 | 1.4 | 1.4 | 1.4 | 1.4 | 1.4 | 1.4 | 1.4 | 1.4 | 1.4 | 1.4 | 1.4 | 1.4 | 1.4 | 1.4 | 1.3 | 1.4 | 1.2 | 1.5 | 1.6 | 1.7 | 1.6 |
| **4-5** | 6.3 | 6.7 | 7.4 |  | 1.1 | 1.4 | 1.2 | 1.3 | 1.1 | 1.3 | 1.3 | 1.3 | 1.3 | 1.3 | 1.3 | 1.3 | 1.3 | 1.3 | 1.3 | 1.3 | 1.3 | 1.3 | 1.3 | 1.3 | 1.2 | 1.3 | 1.2 | 1.6 | 1.7 | 1.7 | 1.7 |
| **6** | 7.2 | 8.1 | 7.4 | 6.5 |  | 1.3 | 1.3 | 1.3 | 1.2 | 1.4 | 1.3 | 1.3 | 1.3 | 1.3 | 1.3 | 1.3 | 1.4 | 1.4 | 1.3 | 1.3 | 1.3 | 1.3 | 1.3 | 1.3 | 1.3 | 1.4 | 1.3 | 1.5 | 1.7 | 1.7 | 1.6 |
| **7** | 9.1 | 9.5 | 9.3 | 9.3 | 8.8 |  | 1.1 | 1.1 | 1.3 | 1.3 | 1.4 | 1.4 | 1.4 | 1.4 | 1.4 | 1.4 | 1.4 | 1.4 | 1.4 | 1.3 | 1.3 | 1.4 | 1.3 | 1.4 | 1.3 | 1.3 | 1.4 | 1.5 | 1.7 | 1.8 | 1.7 |
| **8** | 7.4 | 7.9 | 7.7 | 7.0 | 7.9 | 6.3 |  | 1.1 | 1.2 | 1.3 | 1.4 | 1.4 | 1.4 | 1.4 | 1.3 | 1.4 | 1.4 | 1.4 | 1.4 | 1.3 | 1.3 | 1.4 | 1.3 | 1.4 | 1.2 | 1.3 | 1.3 | 1.6 | 1.7 | 1.8 | 1.7 |
| **9** | 7.9 | 8.1 | 8.8 | 8.1 | 8.4 | 6.0 | 6.5 |  | 1.2 | 1.2 | 1.3 | 1.3 | 1.3 | 1.3 | 1.3 | 1.3 | 1.3 | 1.3 | 1.3 | 1.3 | 1.3 | 1.3 | 1.3 | 1.3 | 1.2 | 1.2 | 1.2 | 1.6 | 1.7 | 1.7 | 1.7 |
| **10** | 8.6 | 9.3 | 8.6 | 7.0 | 8.1 | 8.8 | 8.1 | 7.9 |  | 1.0 | 1.0 | 1.1 | 1.0 | 1.0 | 1.0 | 1.0 | 1.1 | 1.1 | 1.1 | 1.0 | 1.1 | 1.1 | 1.1 | 1.0 | 1.0 | 1.1 | 1.0 | 1.5 | 1.7 | 1.7 | 1.7 |
| **11** | 8.6 | 8.6 | 9.5 | 8.6 | 9.1 | 9.3 | 8.8 | 7.4 | 4.7 |  | 1.0 | 1.1 | 1.0 | 1.0 | 1.0 | 1.1 | 1.1 | 1.1 | 1.1 | 1.0 | 1.0 | 1.1 | 1.0 | 1.0 | 0.9 | 1.0 | 0.9 | 1.5 | 1.7 | 1.7 | 1.6 |
| **12-13** | 9.5 | 9.3 | 10.0 | 9.5 | 8.4 | 10.0 | 9.5 | 8.8 | 6.0 | 5.6 |  | 0.2 | 0.4 | 0.4 | 0.4 | 0.4 | 0.7 | 0.6 | 0.6 | 0.5 | 0.5 | 0.6 | 0.7 | 0.4 | 1.0 | 1.1 | 1.1 | 1.6 | 1.6 | 1.8 | 1.7 |
| **14-16** | 9.8 | 9.5 | 10.2 | 9.8 | 8.6 | 10.2 | 9.8 | 9.1 | 6.3 | 5.8 | 0.2 |  | 0.3 | 0.3 | 0.4 | 0.4 | 0.7 | 0.6 | 0.6 | 0.5 | 0.5 | 0.6 | 0.6 | 0.3 | 1.0 | 1.1 | 1.2 | 1.6 | 1.6 | 1.8 | 1.7 |
| **17-18** | 9.3 | 9.5 | 9.8 | 9.3 | 8.1 | 9.8 | 9.3 | 9.1 | 5.8 | 5.3 | 0.7 | 0.5 |  | 0.0 | 0.2 | 0.2 | 0.6 | 0.5 | 0.5 | 0.3 | 0.4 | 0.5 | 0.5 | 0.0 | 1.0 | 1.0 | 1.1 | 1.6 | 1.6 | 1.8 | 1.6 |
| **19-21** | 9.3 | 9.5 | 9.8 | 9.3 | 8.1 | 9.8 | 9.3 | 9.1 | 5.8 | 5.3 | 0.7 | 0.5 | 0.0 |  | 0.2 | 0.2 | 0.6 | 0.5 | 0.5 | 0.3 | 0.4 | 0.5 | 0.5 | 0.0 | 1.0 | 1.0 | 1.1 | 1.6 | 1.6 | 1.8 | 1.6 |
| **22** | 9.1 | 9.3 | 9.5 | 9.1 | 7.9 | 10.0 | 9.1 | 8.8 | 5.6 | 5.1 | 0.9 | 0.7 | 0.2 | 0.2 |  | 0.3 | 0.6 | 0.5 | 0.6 | 0.4 | 0.4 | 0.5 | 0.5 | 0.2 | 1.0 | 1.1 | 1.1 | 1.5 | 1.6 | 1.7 | 1.6 |
| **23** | 9.5 | 9.8 | 10.0 | 9.5 | 8.4 | 10.0 | 9.5 | 9.3 | 6.0 | 5.6 | 0.9 | 0.7 | 0.2 | 0.2 | 0.5 |  | 0.6 | 0.6 | 0.6 | 0.4 | 0.5 | 0.5 | 0.6 | 0.2 | 1.0 | 1.0 | 1.2 | 1.6 | 1.6 | 1.8 | 1.7 |
| **24** | 9.5 | 9.8 | 10.0 | 9.8 | 8.6 | 10.2 | 9.5 | 9.3 | 7.0 | 6.0 | 2.3 | 2.1 | 1.6 | 1.6 | 1.9 | 1.9 |  | 0.4 | 0.5 | 0.6 | 0.6 | 0.6 | 0.7 | 0.6 | 1.1 | 1.1 | 1.2 | 1.6 | 1.7 | 1.8 | 1.6 |
| **25-26** | 9.5 | 9.8 | 10.0 | 9.8 | 8.6 | 10.2 | 10.0 | 9.3 | 6.5 | 6.0 | 1.9 | 1.6 | 1.2 | 1.2 | 1.4 | 1.4 | 0.7 |  | 0.4 | 0.5 | 0.5 | 0.5 | 0.7 | 0.5 | 1.0 | 1.1 | 1.2 | 1.6 | 1.7 | 1.7 | 1.6 |
| **27** | 8.8 | 9.1 | 9.8 | 9.1 | 8.4 | 10.0 | 9.8 | 9.1 | 6.3 | 5.8 | 1.6 | 1.9 | 1.4 | 1.4 | 1.6 | 1.6 | 1.4 | 0.7 |  | 0.6 | 0.6 | 0.6 | 0.7 | 0.5 | 1.0 | 1.1 | 1.1 | 1.6 | 1.6 | 1.7 | 1.6 |
| **28-29** | 9.3 | 9.5 | 9.8 | 9.3 | 8.6 | 9.3 | 8.8 | 8.6 | 5.8 | 5.3 | 1.2 | 0.9 | 0.5 | 0.5 | 0.7 | 0.7 | 1.6 | 1.2 | 1.4 |  | 0.2 | 0.5 | 0.5 | 0.3 | 1.0 | 1.0 | 1.1 | 1.6 | 1.6 | 1.7 | 1.6 |
| **30** | 9.1 | 9.5 | 9.8 | 9.5 | 8.8 | 9.5 | 9.1 | 8.4 | 6.0 | 5.6 | 1.4 | 1.2 | 0.7 | 0.7 | 0.9 | 0.9 | 1.9 | 1.4 | 1.6 | 0.2 |  | 0.5 | 0.5 | 0.4 | 1.0 | 1.0 | 1.1 | 1.6 | 1.6 | 1.7 | 1.6 |
| **31** | 9.5 | 9.8 | 10.0 | 10.0 | 8.4 | 10.2 | 9.8 | 9.3 | 6.3 | 6.0 | 1.9 | 1.6 | 1.2 | 1.2 | 1.4 | 1.4 | 1.9 | 1.4 | 1.6 | 1.2 | 1.4 |  | 0.6 | 0.5 | 1.0 | 1.0 | 1.1 | 1.6 | 1.6 | 1.7 | 1.6 |
| **32** | 8.4 | 8.8 | 9.1 | 9.3 | 8.6 | 9.8 | 8.8 | 8.1 | 6.3 | 5.8 | 2.1 | 1.9 | 1.4 | 1.4 | 1.2 | 1.6 | 2.6 | 2.1 | 2.3 | 1.4 | 1.2 | 2.1 |  | 0.5 | 1.0 | 1.0 | 1.1 | 1.6 | 1.6 | 1.7 | 1.6 |
| **33** | 9.3 | 9.5 | 9.8 | 9.3 | 8.1 | 9.8 | 9.3 | 9.1 | 5.8 | 5.3 | 0.7 | 0.5 | 0.0 | 0.0 | 0.2 | 0.2 | 1.6 | 1.2 | 1.4 | 0.5 | 0.7 | 1.2 | 1.4 |  | 1.0 | 1.0 | 1.1 | 1.6 | 1.6 | 1.8 | 1.6 |
| **34-35** | 7.7 | 8.1 | 7.9 | 7.9 | 8.4 | 8.1 | 7.9 | 7.9 | 4.7 | 4.2 | 5.1 | 5.3 | 4.9 | 4.9 | 4.7 | 5.1 | 6.0 | 5.6 | 5.3 | 4.9 | 5.1 | 5.6 | 5.3 | 4.9 |  | 1.0 | 0.8 | 1.5 | 1.7 | 1.7 | 1.7 |
| **36** | 8.4 | 8.6 | 9.5 | 9.1 | 9.1 | 8.4 | 8.6 | 7.0 | 6.0 | 4.7 | 6.0 | 5.8 | 5.3 | 5.3 | 5.6 | 5.6 | 6.5 | 6.0 | 6.3 | 5.1 | 4.9 | 5.6 | 5.6 | 5.3 | 5.1 |  | 1.0 | 1.5 | 1.6 | 1.7 | 1.7 |
| **37** | 7.4 | 7.7 | 7.2 | 8.4 | 7.7 | 9.5 | 9.1 | 8.4 | 5.1 | 4.2 | 6.0 | 6.3 | 5.8 | 5.8 | 5.6 | 6.0 | 6.5 | 6.0 | 5.8 | 5.8 | 5.8 | 5.6 | 6.0 | 5.8 | 3.3 | 4.9 |  | 1.4 | 1.6 | 1.6 | 1.6 |
| **38** | 12.8 | 12.8 | 12.8 | 12.6 | 12.3 | 13.0 | 14.0 | 13.3 | 11.9 | 11.6 | 13.0 | 13.3 | 13.3 | 13.3 | 13.0 | 13.5 | 14.0 | 13.7 | 13.5 | 13.5 | 13.7 | 14.0 | 13.5 | 13.3 | 11.6 | 12.1 | 10.9 |  | 1.7 | 1.8 | 1.7 |
| **39** | 15.3 | 15.8 | 15.3 | 15.3 | 15.1 | 17.0 | 16.0 | 16.5 | 15.8 | 15.3 | 15.3 | 15.6 | 15.3 | 15.3 | 15.1 | 15.6 | 16.0 | 15.6 | 14.9 | 15.3 | 15.6 | 15.3 | 15.1 | 15.3 | 15.3 | 15.1 | 13.7 | 14.0 |  | 1.7 | 1.7 |
| **40** | 17.2 | 17.0 | 17.2 | 17.2 | 16.3 | 18.6 | 18.8 | 17.4 | 16.7 | 15.8 | 16.5 | 16.5 | 16.5 | 16.5 | 16.3 | 16.7 | 17.0 | 16.7 | 16.7 | 16.5 | 16.3 | 16.5 | 15.8 | 16.5 | 16.3 | 15.3 | 14.9 | 17.0 | 16.7 |  | 1.6 |
| **41** | 15.6 | 15.3 | 14.7 | 15.8 | 13.7 | 14.9 | 15.8 | 14.2 | 15.1 | 13.0 | 14.7 | 14.4 | 14.4 | 14.4 | 14.2 | 14.7 | 14.7 | 14.7 | 14.9 | 14.4 | 14.2 | 14.2 | 14.0 | 14.4 | 14.0 | 14.4 | 13.0 | 14.4 | 15.3 | 13.0 |  |
