# Supplementary material for: Confirmation of Leptobrachellaventripunctata (Fei, Ye, and Li, 1990), based on molecular and morphological evidence in Thailand
Source: Biodivers Data J. 2021 Oct 14;9:e74097. doi: 10.3897/BDJ.9.e74097 (PMC8530995; doi:10.3897/BDJ.9.e74097)
Supplement: Supplementary material 3 — Measurement and proportions [file bdj-09-e74097-s003.docx]

**Supplementary materials 3.** Measurement (in mm) of and proportions of *Leptobrachella ventripunctata* (see Materials and Methods section for list of abbreviations).

| **Characters** | **AUP-00326** | **Ratio (-/SVL)** |
| --- | --- | --- |
| Sex | Male |  |
| SVL | 28.9 |  |
| HL | 13.1 | 45.3% |
| HW | 11.6 | 40.1% |
| SL | 5.3 | 18.3% |
| ED | 4.6 | 15.9% |
| N-EL | 2.5 | 8.7% |
| SN | 2.7 | 9.3% |
| IND | 3.1 | 10.7% |
| IOD | 3.5 | 12.1% |
| UEW | 4.5 | 15.6% |
| FLL | 18.0 | 62.3% |
| LAL | 15.0 | 51.9% |
| HAL | 7.1 | 24.6% |
| 1FL | 1.3 | 4.5% |
| IPTL | 1.6 | 5.5% |
| OPTL | 1.0 | 3.5% |
| 3FDD | 0.5 | 1.7% |
| HLL | 45.1 | 156.1% |
| TL | 16.2 | 56.1% |
| FL | 14.9 | 51.6% |
| IMTL | 0.9 | 3.1% |
| 1TOEL | 2.4 | 8.3% |
| 4TDD | 0.6 | 2.1% |
| TD | 2.4 | 8.3% |
| OMTL | 1.0 | 3.5% |
